# rs62139665 Polymorphism in the Promoter Region of EpCAM Is Associated With Hepatitis C Virus-Related Hepatocellular Carcinoma Risk in Egyptians

**DOI:** 10.3389/fonc.2021.754104

**Published:** 2022-01-05

**Authors:** Tarek Mohamed Kamal Motawi, Nermin Abdel Hamid Sadik, Dina Sabry, Sally Atef Fahim, Nancy Nabil Shahin

**Affiliations:** ^1^ Department of Biochemistry, Faculty of Pharmacy, Cairo University, Cairo, Egypt; ^2^ Medical Biochemistry and Molecular Biology Department, Faculty of Medicine, Badr University in Cairo, Badr City, Egypt; ^3^ Medical Biochemistry and Molecular Biology Department, Faculty of Medicine, Cairo University, Cairo, Egypt; ^4^ Biochemistry Department, School of Pharmacy, Newgiza University (NGU), Cairo, Egypt

**Keywords:** epithelial cell adhesion molecule, single nucleotide polymorphism, hepatitis C virus, hepatocellular carcinoma, Egyptians

## Abstract

Hepatocellular carcinoma (HCC) is a universal health problem that is particularly alarming in Egypt. The major risk factor for HCC is hepatitis C virus (HCV) infection which is a main burden in Egypt. The epithelial cell adhesion molecule (EpCAM) is a stem cell marker involved in the tumorigenesis and progression of many malignancies, including HCC. We investigated the association of -935 C/G single nucleotide polymorphism in EpCAM promoter region (rs62139665) with HCC risk, EpCAM expression and overall survival in Egyptians. A total of 266 patients (128 HCV and 138 HCC cases) and 117 age- and sex-matched controls participated in this study. Genotyping, performed using allelic discrimination and confirmed by sequencing, revealed a significant association between EpCAM rs62139665 and HCC susceptibility, with higher GG genotype and G allele distribution in HCC patients than in non-HCC subjects. Such association was not detected in HCV patients compared to controls. EpCAM gene expression levels, determined in blood by RT-qPCR, and its serum protein expression levels, determined by ELISA, were significantly higher in GG relative to GC+CC genotype carriers in HCV and HCC patients in a recessive model. ROC analysis of EpCAM protein levels revealed significant discriminatory power between HCC patients and non-HCC subjects, with improved diagnostic accuracy when combining α-fetoprotein and EpCAM compared to that of α-fetoprotein alone. Altogether, EpCAM rs62139665 polymorphism is significantly associated with HCC and with EpCAM gene and protein expression levels in the Egyptian population. Moreover, serum EpCAM levels may hold promise for HCC diagnosis and for improving the diagnostic accuracy of α-fetoprotein.

## 1 Introduction

Hepatitis C virus (HCV) infection is a major burden in Egypt, infecting almost 14.7% of the population. HCV in Egypt is considered the highest worldwide. Chronic HCV is the leading cause of liver-related death in Egypt (1).

Hepatocellular carcinoma (HCC) is considered the third cause of cancer-related mortality globally (2). This high mortality rate could be attributed to the late manifestation of HCC symptoms and, hence, its late diagnosis. Such diagnostic inadequacy is particularly prominent in lower resource settings with limited screening tools (3). Approximately 60–80% of HCV patients develop chronic hepatitis, of which 10–20% develop cirrhosis within 20–30 years. About 1–5% of cirrhotic patients may develop HCC (4). Thus, the presence of cirrhosis increases the risk for HCC, however, some patients develop HCC in non-cirrhotic livers and in the absence of inflammation (5). Several studies reported some genetic and epigenetic defects that lead to the onset of HCC (6, 7).

A minor fragment of cancer stem cells (CSCs) are responsible for the tumor initiation, growth, metastasis and relapse after treatment (8, 9). CSCs are mainly responsible for high resistance to both radiation and chemotherapy (10, 11). The epithelial cell adhesion molecule (EpCAM) is a ~ 40kDa transmembrane glycoprotein located on chromosome 2p21, highly expressed in most epithelial cancers except squamous, urothelial and renal cell carcinomas (12). EpCAM is considered as an important marker for hepatic CSCs (13) and it becomes absent once cells are differentiated into mature hepatocytes (14). Poor prognosis was observed in carcinomas with high EpCAM expression (15). The role of EpCAM is not only restricted to cell-cell linkage, but also plays an important role in migration, proliferation, signaling, differentiation, metastasis and renewal of hepatic cells (16). EpCAM acts by the activation of Wnt signaling and increasing the c-Myc expression in highly proliferating tumor cells (17, 18).

Single nucleotide polymorphisms (SNPs) are considered the most common form of genetic diversity scattered throughout the human genome and is responsible for most variabilities in genetic traits between patients as disease vulnerability, prognosis and response to therapy (19). Promoter region SNPs of a gene regulate its expression since transcription factors bind to certain nucleotide sequences within this region, thus modulating translation and predisposing an individual to certain diseases including cancer (20–22). For example, SNP rs1126497 in EpCAM gene is significantly associated with an increased risk of breast cancer and cervical cancer, as well as the overall survival (OS) of non-small cell lung cancer patients and HCC patients who had portal vein tumor thrombus (19, 23–25). These findings suggest that SNPs in the EpCAM gene may play a significant role in the initiation and progression of various types of cancer.

The objective of the present study was to investigate whether -935 C/G SNP (rs62139665) in the EpCAM gene promoter region is associated with its high expression and, hence, with susceptibility to HCC and OS in HCV Egyptian patients.

## 2 Subjects and Methods

### 2.1 Subjects

The present study was conducted on 266 Egyptian patients categorized into 128 HCV-infected patients and 138 HCV-dependent HCC patients, recruited from the Endemic Medicine and Gastroenterology Department, Faculty of Medicine, Cairo University, from June 2014 until October 2017. The patients were followed-up for 2 years unless they died. Hepatitis C viral RNA was detected in all HCV patients, while HCC patients had HCV infection that was detected by testing positive for anti-HCV antibodies. The medical history of HCC patients is shown in **Table S1**. HCC patients were diagnosed based on pathology, cytology, ultrasound and computed tomography (CT) imaging, in addition to serum levels of alpha-fetoprotein (AFP). Tumor number, lesion size, macroscopic vascular invasion, the TNM stage, portal vein thrombosis, portal hypertension, as well as brain, chest, and total-body bone CT (to rule out extrahepatic metastases) were also evaluated. Model for end-stage liver disease (MELD) and Child-Pugh scores were also determined. HCC patients receiving radiotherapy or chemotherapy or suffering other types of cancer were excluded from the investigation.

One hundred and seventeen apparently healthy volunteers, age- and gender-matched to the patients, joined the study as controls (**Table 1**). They all showed normal liver function profiles, normal hepatobiliary ultrasound, and negative serological results for viral hepatic and autoimmune diseases, with no previous history of liver disease. Liver ultrasound findings in the studied groups are displayed in **Table S2**.

**Table 1:** Demographic characteristics and laboratory data in the HCV, HCC patients and healthy controls.

	Control (n=117)	HCV (n=128)	HCC (n=138)	*P*-value
**Age (years)**	49.17 ± 18.69	48.29 ± 13.2	52.28 ± 10.29	0.06
**Gender**				
**Male**	62 (52.9%)	76 (59.4%)	83 (60.1%)	0.46
**Female**	55 (47.1%)	52 (40.6%)	55 (39.9%)	
**Hemoglobin**	10.82 ± 1.14	13.89 ± 1.48^†^	12.59 ± 1.67^†‡^	<0.0001
**WBCs (x10^3^)**	4.16 ± 1.12	6.49 ± 1.96^†^	5.73 ± 2.15^†‡^	<0.0001
**Platelets (x10^3^)**	186.4 ± 77.48	238.62 ± 110.22^†^	138.61 ± 62.31^†‡^	<0.0001
**PC (%)**	92.03 ± 5.73	79.72 ± 25.73^†^	76.86 ± 13.05^†^	<0.0001
**PT-INR**	1.09 ± 0.11	1.08 ± 0.11	1.24 ± 0.19^†‡^	0.002
**D Bil (mg/dl)**	0.21 ± 0.13	0.39 ± 0.25^†^	0.51 ± 0.34^†‡^	<0.0001
**T Bil (mg/dl)**	0.91 ± 0.22	0.77 ± 0.3^†^	1.22 ± 0.59^†‡^	<0.0001
**ALT (U/L)**	27.55 ± 6.51	46.89 ± 24.07^†^	59.2 ± 36.13^†‡^	<0.0001
**AST (U/L)**	26.82 ± 6.35	54.65 ± 36.31^†^	70.21 ± 39.11^†‡^	<0.0001
**ALP (U/L)**	78.54 ± 29.51	125.72 ± 67.01^†^	180.89 ± 58.54^†‡^	<0.0001
**Albumin (g/dl)**	4.13 ± 0.58	4.22 ± 0.42	3.36 ± 0.52^†‡^	<0.0001
**Creatinine (mg/dl)**	0.98 ± 0.63	0.88 ± 0.21	0.86 ± 0.22^†^	0.038
**AFP (ng/ml)**	3.95 ± 1.89	6.82 ± 15.81	662.45 ± 1462.1^†‡^	<0.0001

Data are expressed as mean ± SD, or n (%).

Gender data were compared using Chi square (X^2^) test. The rest of the data were analyzed using one-way ANOVA and Tukey’s multiple comparisons test.

^†^Significant difference from the control group.

^‡^Significant difference from the HCV group.

WBCs, white blood cells; PC, prothrombin concentration; PT-INR, prothrombin time-international normalized ratios; D Bil, direct bilirubin; T Bil, total bilirubin; ALT, alanine aminotransferase; AST, aspartate aminotransferase; ALP, alkaline phosphatase; AFP, alpha-fetoprotein.

An informed consent form was signed by all the study participants. The study protocol was approved by the Research Ethics Committee, Faculty of Pharmacy, Cairo University (Permit number: BC 1813) and conformed to the ethical guidelines of the 1975 Helsinki Declaration.

Hepatitis B virus (HBV) or human immunodeficiency virus (HIV) antibodies, diabetes, fatty liver, active schistosomiasis, presence of alcohol or heavy metal in blood were considered as exclusion criteria for the study participants.

### 2.2 Methods

#### 2.2.1 Sample Processing and Laboratory Investigations

Ten milliliter-venous blood specimens were obtained from all enrolled subjects by trained laboratory technicians. The collected samples were aliquoted and processed as previously described (22). Briefly, one aliquot was used for RNA and DNA extraction and subsequent gene expression analysis, genotyping and sequencing. A second aliquot was separated into plasma and assayed for albumin as well as prothrombin time-international normalized ratio (PT-INR). A third aliquot was used for serum separation for the assessment of HCV-RNA and antibody titres, AFP and EpCAM levels, alanine aminotransferase (ALT), aspartate aminotransferase (AST) and ALP activities, as well as total and direct bilirubin. The extent of cirrhosis in HCC was evaluated on the basis of the Child scoring system that depends on albumin, bilirubin, prothrombin time, ascites and encephalopathy. Child-Pugh grades were assigned to patients according to Child and Turcotte (26).

#### 2.2.2 Designing Primers and Probes

The EpCAM sequence was acquired from the NCBI. Ensembl genome browser 90 was used to display all variants in order to design primers that do not superimpose SNPs. Then, allele-specific primers and probes were designed using Primer3Plus, and their specificity was checked by Blast and MFEprimer-2.0. We chose a SNP, rs62139665, in the 5’UTR with a MAF exceeding 20% and predicted to modulate the promoter binding affinity to various transcription factors, thus modifying EpCAM gene expression.

#### 2.2.3 EpCAM mRNA Expression Analysis

RNA extraction, reverse transcription and qPCR were performed as previously described (22). Briefly, total RNA extraction from blood samples was performed using a total RNA purification kit (Jena Bioscience, Munich, Germany) followed by storage of the isolated RNA at −80°C until analysis. Reverse transcription was performed using cDNA archive kit (Applied Biosystems, Foster City, California, USA). Quantitative real-time PCR (qRT-PCR) was performed using GoTaq PCR master mix (Promega Co., Madison, USA); 1 µL of cDNA was added to 25 µL of master mix, 0.25 µL of CXR Reference Dye, 1 µL of forward and reverse primers and the volume was completed to 50 µL. A protocol comprising an initial denaturation step at 95°C for 10 minutes, followed by 40 cycles of denaturation at 95°C for 15 seconds, and annealing and extension at 60°C for 1 minute, then 60°C for 30 seconds was conducted on a 7500 Real-Time PCR system (Applied Biosystems, Foster City, California, USA). The used primers had the following sequences: 5′- AGTGTAATGGCACGATCTCTG -3’ (forward), 5’-GGATCACCTGAGGTTTGAAGT -3’ (reverse) for EpCAM, with β-actin as an internal control.

#### 2.2.4 Genotyping of EpCAM Single Nucleotide Polymorphism rs62139665

DNA extraction and genotyping were carried out as formerly detailed (22), using the primers: 5’- AGTGTAATGGCACGATCTCTG -3’ (forward) and 5’- GGATCACCTGAGGTTTGAAGT -3’ (reverse), and the two tagged probes: VIC-TAGTAGAGACGGGGTTCCTCCATGT and FAM- TAGTAGAGACGGCGTTCCTCCATGT. 6-carboxy-X-rhodamine (ROX) was used as a passive reference dye.

#### 2.2.5 Sanger Sequencing

To verify the allelic discrimination results, twenty samples from each genotype were sequenced as previously described by Motawi and co-workers (22). The used primer sequences were as follows: 5’- GGCTCTATGGGAACACCTTT -3’ (forward) and 5’- GGATCACCTGAGGTTTGAAGT -3’ (reverse). The amplicon size was 240 bp.

#### 2.2.6 Determination of Serum EpCAM Levels

Serum EpCAM protein concentration was determined by an ELISA kit supplied by Boster Biological Technology (Catalogue no. EK0755, Pleasanton, CA, USA) in compliance with its operational guidelines.

#### 2.2.7 Statistical Analysis

Data are presented as mean ± SD, number (percentage) or median (interquartile range). The differences between two groups were statistically analyzed by Student’s t-test and Chi square test for numerical and categorical variables, respectively. The variations between the three groups were assessed using one-way ANOVA followed by Tukey’s multiple comparisons *post-hoc* test. Receiver operating characteristic (ROC) analysis was performed to calculate EpCAM and AFP sensitivity and specificity, individually or in combination. The correlation between EpCAM and AFP levels was tested by Spearman’s correlation analysis. Four models (dominant, recessive, overdominant and multiplicative) were used to assess the association between each genotype and the risk of HCC. Logistic regression was conducted to estimate the odds ratios (ORs) and 95% confidence intervals (CIs) of the association between EpCAM SNP rs62139665 and HCC risk. For a two-tailed test, a *P*-value lower than 0.05 was considered statistically significant. The Kaplan–Meier method and the log-rank survival test were employed to estimate the OS. The GraphPad Prism 6 (GraphPad Software, CA, USA) and the SPSS software, version 20.0 (SPSS Inc. Chicago, IL, USA) statistical packages were used to perform the statistical analyses. Hardy-Weinberg equilibrium (HWE) was tested online (http://www.oege.org/software/hwe-mr-calc.shtml).

## 3 Results

### 3.1 Demographic, Laboratory and Clinical Characteristics of the Study Participants

The demographic features as well as the laboratory and clinical data of the study participants are depicted in **Tables 1**, **S1** and **S2**. Neither age nor gender varied significantly between the studied groups.

### 3.2 Genotype Distribution and Allele Frequencies of EpCAM rs62139665 Polymorphism in the Studied Groups, and Compliance With Hardy-Weinberg Equilibrium

The genotype frequencies in the control, HCV and HCC groups were in agreement with the assumption of a Hardy–Weinberg equilibrium (*P* > 0.05) as displayed in **Table 2**.

**Table 2:** Hardy Weinberg equilibrium for EpCAM -935 C/G (rs62139665).

Group	Observed frequency			Expected frequency			*P*-value
Genotype	CC	GC	GG	CC	GC	GG	
**Control**	45	60	12	48.08	53.85	15.08	0.22
**HCV**	58	56	14	57.78	56.44	13.78	0.93
**HCC**	35	69	34	35	69	34	0.99

The Chi square test was used to determine deviation from Hardy-Weinberg equilibrium (HWE).

**Figure 1:**
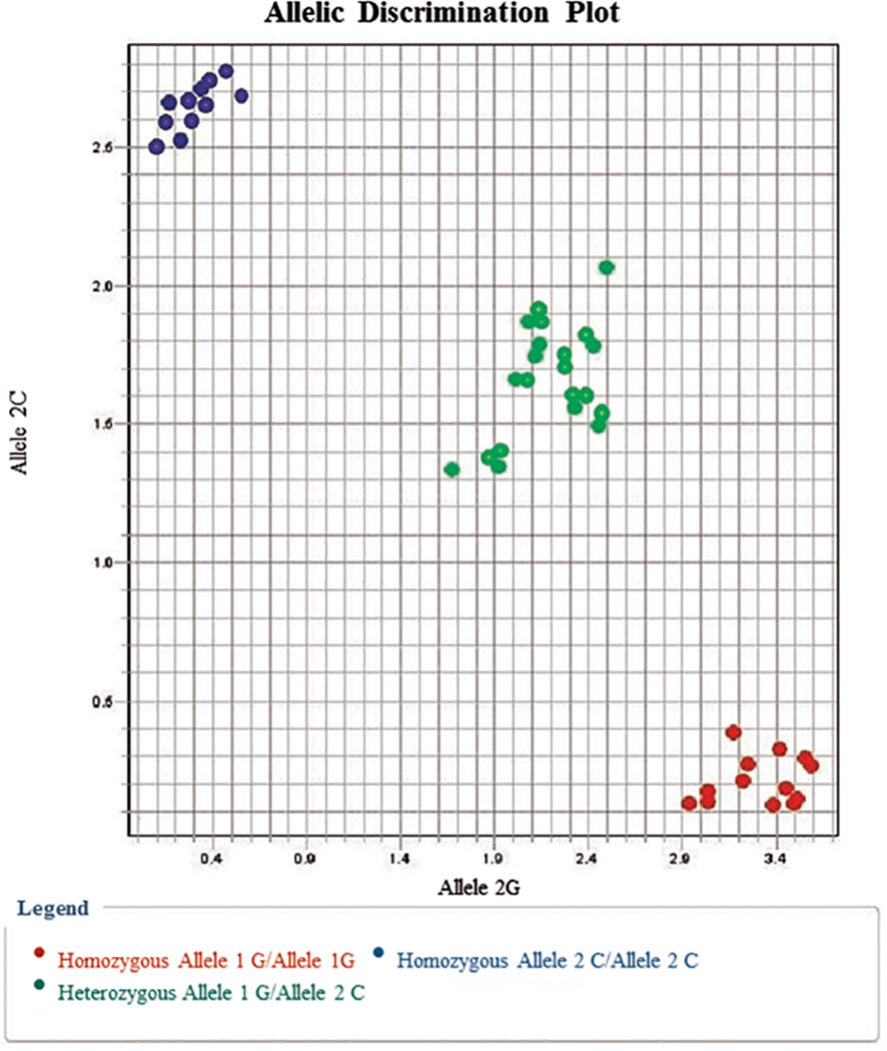
Allelic discrimination plot for EpCAM rs62139665 G/C alleles showing the C/C genotype (represented by upper left dots) near the Y-axis, the G/G genotype (represented by lower right dots) near the X-axis, and the G/C genotype in the middle between both axes.

**Table 3** and **Figure 1** illustrate the genotype and allele frequencies of EpCAM rs62139665 polymorphism. The GG genotype and G allele frequencies were significantly higher in the HCC group compared to the HCV patients (*P* = 0.0005 and *P* < 0.0001, respectively) and to the control subjects (*P* = 0.04 and *P* = 0.001, respectively), while no significant difference was found between HCV and control groups. According to the genetic model selection strategy (27), a recessive model was chosen as it best fits the analysis of the association between rs62139665 and HCC risk. **Table 4** depicts the association of rs62139665 with HCV and HCC. In HCC cases, the rs62139665 GG genotype carriers displayed a markedly higher distribution than GC+CC genotype carriers relative to the control (OR = 2.86, *P =* 0.003) and HCV (OR = 2.66, *P* = 0.004) groups. Furthermore, the HCC cases exhibited an appreciably higher G allele frequency than in the control (OR = 1.76, *P* = 0.002), and HCV (OR = 2.02, *P* = 0.0001) groups.

**Table 3:** Frequency distribution for genotypes and alleles for EpCAM rs62139665 in patients and control groups.

	Control	HCV	HCC	
**CC**	45 (0.39)	58 (0.45)	35 (0.25)	
**GC**	60 (0.51)	56 (0.44)	69 (0.5)	
**GG**	12 (0.1)	14 (0.11)	34 (0.25)	
**P-value**	0.04^†^	0.48^‡^	0.0005^§^	0.0006^¶^
**C**	150 (0.64)	172 (0.67)	139 (0.51)	
**G**	84 (0.36)	84 (0.33)	137 (0.49)	
**P-value**	0.001^†^	0.47^‡^	<0.0001^§^	0.0001^¶^

Data are expressed as N (%).

^†^χ^2^ test for difference in the frequency in HCC vs control.

^‡^χ^2^ test for difference in the frequency in HCV vs control.

^§^χ^2^ test for difference in the frequency in HCC vs HCV.

^¶^χ^2^ test for difference in the frequency in the study population.

**Table 4:** Genotype and allele frequency of EpCAM -935 C/G polymorphism in different study groups and its association to HCV and HCC risk by logistic regression analysis.

	Genotype/Allele	Control (117) N(%)	HCV (128) N(%)	HCC (138) N(%)	OR^†^ (95% CI)	*P*-Value^†^	OR^‡^ (95% CI)	*P*-Value^‡^	OR^§^ (95% CI)	*P*-Value^§^
**Codominant**	CC	45	58	35	Ref		Ref		Ref	
		(38.5%)	(45.3%)	(25.4%)						
	GC	60	56	69	1.47	0.2	0.72	0.28	2.04	0.01
		(51.3%)	(43.8%)	(50%)	(0.84-2.59)		(0.42-1.23)		(1.18-3.53)	
	GG	12	14	34	3.64	0.001	0.9	0.83	4.02	0.0002
		(10.2%)	(10.9%)	(24.6%)	(1.64-8.04)		(0.38-2.15)		(1.89-8.52)	
**Dominant**	GC + GG	72	70	103	1.84	0.03	0.75	0.3	2.43	0.0008
		(61.5)	(54.7%)	(74.6%)	(1.07-3.14)		(0.45-1.25)		(1.45-4.09)	
**Recessive**	GC + CC	105	114	104	2.86	0.003	1.07	1	2.66	0.004
		(89.7%)	(89.1%)	(75.4%)	(1.4-5.83)		(0.47-2.43)		(1.35-5.23)	
**Overdominant**	CC+GG	57	72	69	1.05	0.9	1.35	0.25	0.78	0.32
		(48.7%)	(56.2%)	(50%)	(0.64-1.72)		(0.81-2.24)		(0.47-1.26)	
**Alleles**	C	150	172	139	Ref		Ref		Ref	
		(64.1%)	(67.2%)	(50.4%)						
	G	84	84	137	1.76	0.002	0.87	0.5	2.02	0.0001
		(35.9%)	(32.8%)	(49.6%)	(1.23-2.51)		(0.6-1.26)		(1.41-2.86)	

The odds ratios (ORs) and confidence intervals (CIs) of 95 percent were estimated by logistic regression for association analysis.

^†^HCC vs Control.

^‡^HCV vs Control.

^§^HCC vs HCV.

HCC, hepatocellular carcinoma; HCV, hepatitis C virus; OR, odds ratio.

The results reported herein accentuate a notable association between EpCAM rs62139665 SNP and HCC susceptibility in Egyptians. Nevertheless, EpCAM rs62139665 was not significantly linked with HCV risk in the tested sample.

### 3.3 Association of EpCAM rs62139665 Polymorphism With the Clinicopathological Features of HCC Patients

**Figure 2:**
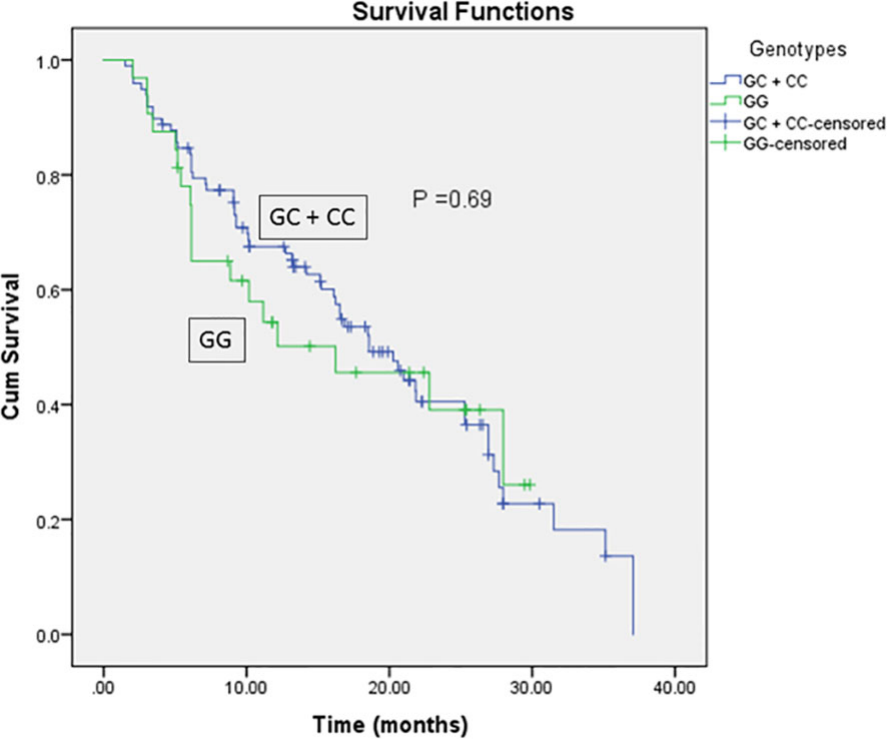
Kaplan-Meier and Log-rank survival curves for HCC patients in relation to EpCAM rs62139665 genotypes in a recessive model.

As for the clinicopathological variables, the GG genotype was significantly correlated with higher ALP activity compared to GC + CC genotypes (*P* = 0.01, **Table 5**). In addition, the Kaplan-Meier and log-rank survival tests showed insignificantly lower overall survival and survival time in EpCAM rs62139665 GG genotype carriers compared with GC + CC genotype carriers in the HCC group. Moreover, the GC + CC genotype carriers exhibited non-significantly lower MELD score when compared with the GG genotype carriers (**Table 5** and **Figure 2**).

**Table 5:** The associations of the biochemical parameters and the EpCAM -935 C/G polymorphism in HCC patients.

Genotype/Parameter	GC + CC (n = 104)	GG (n = 34)	*P*-Value
**Hemoglobin**	12.57 ± 1.58	12.67 ± 1.93	0.75
**WBCs (x10^3^)**	5.82 ± 1.99	5.45 ± 2.62	0.39
**Platelets (x10^3^)**	142 ± 61.18	128.3 ± 65.54	0.27
**PC (%)**	77.42 ± 13.61	75.16 ± 11.23	0.38
**PT-INR**	1.23 ± 0.21	1.26 ± 0.14	0.37
**D Bil (mg/dl)**	0.5 ± 0.37	0.54 ± 0.27	0.58
**T Bil (mg/dl)**	1.21 ± 0.6	1.27 ± 0.58	0.6
**ALT (U/L)**	56.42 ± 34.97	67.82 ± 38.81	0.11
**AST (U/L)**	67.45 ± 38.25	67.73 ± 33.78	0.59
**ALP (U/L)**	173.7 ± 56.29	205 ± 60.56	0.01*
**Albumin (g/dl)**	3.33 ± 0.56	3.46 ± 0.39	0.23
**Creatinine (mg/dl)**	0.86 ± 0.23	0.86 ± 0.19	0.99
**Survival period (months)**	15.31 ± 9.15	13.03 ± 8.87	0.22
**MELD score**	9 (6-11)	10 (6-15)	0.2
**Child–Pugh score**	***A* (n=68); *B* (n=30); *C* (n=6)**	***A* (n=25); *B* (n=9); *C* (n=0)**	0.32
**AFP (ng/ml)**	238.4 ± 430	1947 ± 2445	<0.0001*

Data are expressed as mean ± SD or median (interquartile range), P < 0.05 was significant. Data were compared using Student’s t-test for parametric tests, Mann‐Whitney test for non-parametric tests and Chi square test for categorical variables.

WBCs, white blood cells; PC, prothrombin concentration; PT-INR, prothrombin time-international normalized ratios; D Bil, direct bilirubin; T Bil, total bilirubin; ALT, alanine aminotransferase; AST, aspartate aminotransferase; ALP, alkaline phosphatase; MELD, model for end‐stage liver disease; A, B and C, Child-Pugh grades A (5 to 6 points, B (7 to 9 points) and C (10 to 15 points) according to the criteria indicated by Child and Turcotte (26); AFP, alpha-fetoprotein.

### 3.4 EpCAM Gene and Protein Expression Levels in the Studied Groups

As illustrated in **Table 6**, the expression of EpCAM at both the gene and protein levels was notably higher in HCV (*P* < 0.0001 and *P* < 0.05, respectively) and HCC (*P* < 0.0001) patients compared to the control group. Moreover, EpCAM protein expression level was appreciably higher in HCC than in HCV patients (*P* < 0.0001), whereas the gene expression level did not vary significantly between the two groups. Comparing the expression of EpCAM between rs62139665 genotypes, we observed a significant link between EpCAM rs62139665 polymorphism and its expression. The GG carriers exhibited significantly higher EpCAM gene and protein expression levels compared to the GC + CC carriers in each of the three studied groups (*P* < 0.05 and *P* < 0.0001 regarding gene and protein expression, respectively, within the control group; *P* < 0.0001 for both gene and protein expression within each of the HCV and HCC groups).

**Table 6:** Gene and protein expression levels of EpCAM in different genotypes of rs62139665.

Groups	Genotype	Gene expression levels		Protein expression levels	
**Control**	**GG**	2.71 ± 0.42	3.28 ± 0.75	362.8 ± 17.59	281.2 ± 75.3
	**GC + CC**	3.85 ± 0.54^§*^		247.2 ± 62.54^§**^	
**HCV**	**GG**	1.01 ± 0.21	1.66 ± 0.74^†**^	819.7 ± 84.05	440.8 ± 247.6^†*^
	**GC + CC**	2.08 ± 0.66^§**^		356.7 ± 183.4^§**^	
**HCC**	**GG**	0.95 ± 0.35	1.59 ± 0.56^†**^	1046 ± 115.1	798.5 ± 243.2^†**‡**^
	**GC + CC**	1.95 ± 0.24^§**^		637.6 ± 148.4^§**^	

EpCAM gene expression is expressed as ΔCt mean ± SD, where ΔCt = Ct value of EpCAM - Ct value of β-actin; a smaller ΔCt value corresponds to a higher gene expression level.

EpCAM protein expression is expressed as mean ± SD.

The data were analyzed using Student’s t-test for comparing 2 groups and one-way ANOVA followed by Tukey’s multiple comparisons test for comparing the three studied groups.

^†^Significant difference from the control group.

^‡^Significant difference from the HCV group.

^§^Significant difference from GG within the same group.

*Significant at P < 0.05.

**Significant at P < 0.0001.

### 3.5 Serum AFP Level, Correlation With EpCAM Level, and ROC Analysis of Discriminatory Performance of AFP and EpCAM Individually and in Combination

Comparing the serum AFP levels in the three studied groups revealed markedly higher levels in the HCC group than in each of the control and HCV groups (*P* < 0.0001, **Table 1**). As illustrated in **Figure S1**, a strong positive correlation was observed between AFP and EpCAM protein levels (r = 0.99, *P* < 0.0001).

The ROC curves of EpCAM protein expression level depict significant discriminatory power between HCC patients and non-HCC subjects (AUC = 0.92, CI = 0.87-0.97, *P* < 0.0001), as shown in **Figure S2**, suggesting comparable diagnostic accuracy to that of AFP (AUC = 0.961, CI = 0.92-0.99, *P* < 0.0001). Combining AFP and EpCAM led to an improved diagnostic accuracy than that of either AFP or EpCAM alone (AUC = 0.99, CI = 0.95-1, *P* < 0.0001).

### 3.6 Corroboration of EpCAM rs62139665 Genotyping Findings by Sanger Sequencing

**Figure 3:**
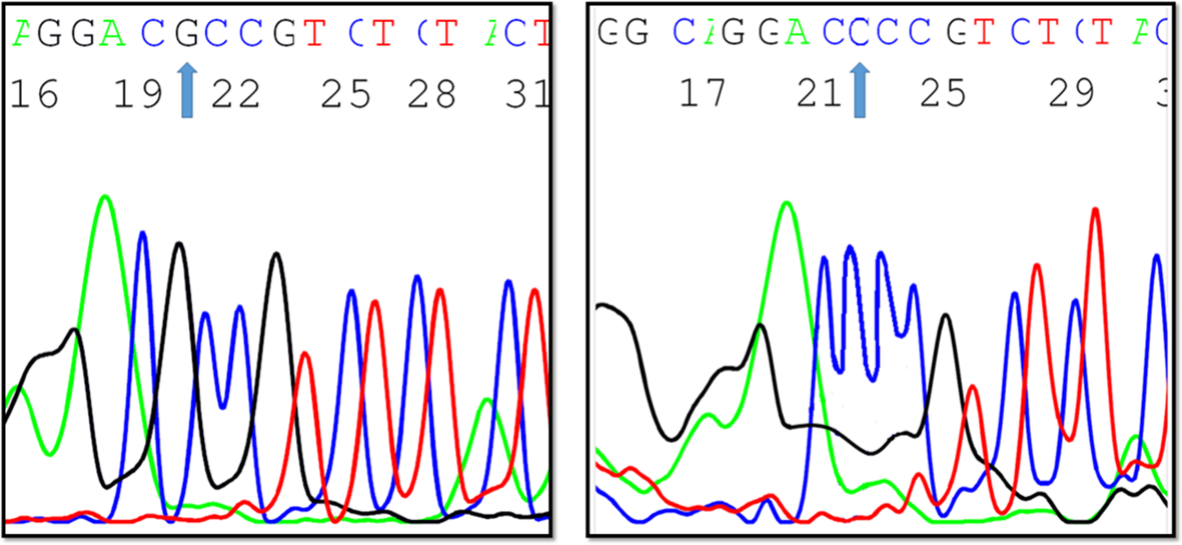
A Sanger sequencing chromatogram depicting EpCAM -935 C/G variants (created by ABI Genetic Analyzer).

Upon comparing the findings of EpCAM rs62139665 genotyping obtained by allelic discrimination with those of Sanger sequencing, both outcomes were perfectly matched for all the genotypes in the tested samples (**Figure 3**).

## 4 Discussion

Worldwide, Egypt endures the highest incidence of HCV infection that is considered a main predisposing factor for the progression of HCC. This investigation was undertaken to explore the association of -935 C/G (rs62139665) SNP in the promoter region of EpCAM with the risk of HCV-related HCC, EpCAM expression levels and the OS in the Egyptian population.

The present investigation demonstrates, for the first time, the association of EpCAM rs62139665 with HCC risk. rs62139665 is located at 935 upstream in the promoter region and it was reported that the transcriptional activity of 1.1kb upstream of the EpCAM gene is closely associated with the levels of EpCAM 28. Mutations in the EpCAM gene were reported in patients having Lynch syndrome through deletions in the 3’UTR 29 or congenital tufting enteropathy that results in decreasing EpCAM protein level (28).

In the current study, we adopted the allelic discrimination method to examine -935 C/G gene polymorphism (rs62139665) in the promoter region of EpCAM, and we, thereafter, verified the results by direct DNA sequencing. The genotype distribution in the three studied groups agreed with HWE.

It is worthy to note that there is a variation in the incidence of EpCAM rs62139665 SNP that is related to ethnicity, as reported in the NCBI map database for diverse ethnic populations, revealing C and G allele frequencies of C = 0.16 and **G** = 0.83 in African Carribbeans, C = 0.17 and **G** = 0.83 in Han Chinese and **C** = 0.64 and G = 0.36 in Toscani in Italia. The EpCAM rs62139665 C and G allele frequencies disclosed herein in the control group were 0.64 and 0.36, respectively, which are quite in accordance with those reported in the Toscani Italian population, having the C allele as the major allele.

The SNP rs62139665 distribution was significantly different between HCC patients and non-HCC subjects at both genotype and allelic levels. The EpCAM GG genotype and G allele were significantly more frequent in HCC patients than in HCV patients and controls, proposing that the presence of the C allele may have protective effects. By using logistic regression analyses, we found a significant association between HCC risk and the rs62139665 G allele and GG genotype when compared to GC + CC in a recessive model. This association with HCC risk can be explained and evidenced by the significant up-regulatory effect of the GG genotype on EpCAM gene and protein expression levels observed in the present study. Numerous studies showed that SNPs in the promoter region are related to increased gene expression (22, 29, 30).

The HCV group displayed significantly higher gene and protein expression of EpCAM compared to the control group despite the lack of significant difference in rs62139665 genotype and allele frequencies between the two groups. The higher expression of EpCAM observed in the HCV group compared to the control could be linked to the reported HCV-induced elevation of plasminogen activator inhibitor-1 (PAI-1). Increased expression of PAI-1 subsequent to HCV infection promoted the cancer stem-like cells (CSC) state in HCV-infected hepatocytes through the activation of the chief mediator of cell proliferation, protein kinase B, in consort with the increased expression of the CSC marker, EpCAM (31).

Several studies reported the role of EPCAM gene in HCC pathogenesis, as a prognostic marker and in HCC recurrence (32–35). Yet, referring to the TCGA and GEO databases (data compared by GEO2R), it was found that EPCAM gene showed non-significant increase in expression in HCC samples compared to their normal controls (GSE49515), and in HCC patients with and without venous metastasis (GSE5093).

Former studies reported significant association of EpCAM expression in HCC with high tumor grade and AFP level (36, 37). In our current study, the expression levels of AFP and EpCAM were significantly higher in the HCC group relative to the HCV and control groups. Furthermore, a positive correlation was found between AFP and EpCAM protein levels. These results are in line with other published data that also reported the overexpression of EpCAM in cirrhotic and liver tumor tissues compared to normal tissues (38). In addition, AFP level can predict the expression of hepatic progenitor cell markers as EpCAM in HCC (39) and is correlated with tumor metastasis (40). ROC analysis was conducted in the present study to compare the diagnostic performance of AFP and EpCAM. It showed that a combined model of both AFP and EPCAM had a higher specificity than either of them alone at all levels of sensitivity. Previously, AFP^+^/EpCAM^+^ HCC was characterized by poorer prognosis compared to AFP^−^/EpCAM^−^ HCC (35).

By examining the influence of the SNP rs62139665 on the disease outcomes, through correlating the SNP rs62139665 genotypes with several parameters as liver function tests, a significant correlation was detected only between GG genotype frequency, hence high EpCAM expression, and ALP activity. Such association may be attributed to ALP being an embryonic stem cell marker that has been observed during cell reprogramming (41). Furthermore, the inhibition of EpCAM expression resulted in diminished ALP activity (42).

Both OS and the survival period of rs62139665 GG genotype carriers were shorter compared with the GC + CC genotype carriers in HCC patients but did not reach statistical significance. Given the overexpression of EpCAM observed in GG genotype carriers compared to GC + CC genotype carriers, our finding of apparently shorter OS in GG genotype carriers compared with the GC + CC genotype carriers is in accordance with Noh and co-workers’ suggestion that patients with positive immunohistochemical expression of EpCAM had reduced OS compared to those who were EpCAM-negative after undergoing surgical resection for HCC (43). Moreover, the detection of EpCAM-positive circulating tumor cells is strongly correlated with the clinical outcome and OS in patients with HCC (39).

In the current study, the MELD score showed no significant difference between GC + CC and GG genotype carriers. Our observation seems to be in agreement with Sancho-Bru and co-workers’ report of lack of correlation between EpCAM gene expression and MELD score in patients with alcoholic hepatitis (44). On the other hand, patients with liver cirrhosis who were infused with EpCAM-positive stem cells showed a significant decrease in MELD score and a marked clinical improvement.

In conclusion, the present study accentuates the association of EpCAM rs62139665 SNP, specifically the G allele frequency, with HCC risk. However, no such association was found with HCV infection in the tested sample of Egyptians. In addition, the present findings highlight the association of the rs62139665 GG genotype with increased EpCAM expression at both the gene and protein levels. Further research on larger datasets, other ethnicities and other cancer types are warranted for a comprehensive elucidation of the associations of EpCAM -935 C/G polymorphism with cancer risk.

## Data Availability Statement

The datasets presented in this study can be found in online repositories. The name of the repository and accession numbers can be found below: National Center for Biotechnology Information (NCBI) GenBank, https://www.ncbi.nlm.nih.gov/genbank/, MZ826468, MZ826469, MZ826470 and MZ826471.

## Ethics Statement

The studies involving human participants were reviewed and approved by The Research Ethics Committee, Faculty of Pharmacy, Cairo University (Permit number: BC 1813). The patients/participants provided their written informed consent to participate in this study.

## Author Contributions 

TM and NAHS conceived, designed and coordinated the study and revised the manuscript. DS collected the samples and participated in the experimental design and lab work. SF carried out the lab work and the statistical analyses and drafted the manuscript. NNS participated in the experimental design, data analysis and curation, and revised and edited the manuscript. All authors contributed to the article and approved the submitted version.

## Funding

The present work has been partially funded by the Faculty of Pharmacy, Cairo University (Cairo, Egypt).

## Conflict of Interest

The authors declare that the research was conducted in the absence of any commercial or financial relationships that could be construed as a potential conflict of interest.

## Publisher’s Note

All claims expressed in this article are solely those of the authors and do not necessarily represent those of their affiliated organizations, or those of the publisher, the editors and the reviewers. Any product that may be evaluated in this article, or claim that may be made by its manufacturer, is not guaranteed or endorsed by the publisher.

## Abbreviations

AFP, Alpha-fetoprotein; ALP, Alkaline phosphatase; ALT, Alanine aminotransferase; AST, Aspartate aminotransferase; CIs, confidence intervals; CSCs, Cancer stem cells; CT, Computed tomography; EpCAM, Epithelial cell adhesion molecule; HCC, Hepatocellular carcinoma; HCV, Hepatitis C virus; HWE, Hardy-Weinberg equilibrium; MELD, Model for end-stage liver disease; OR, odds ratios; OS, Overall survival; PT-INR, Prothrombin time-international normalized ratio; ROC, Receiver operating characteristic; SNPs, Single nucleotide polymorphisms.
