# Supplementary material for: rs62139665 Polymorphism in the Promoter Region of EpCAM Is Associated With Hepatitis C Virus-Related Hepatocellular Carcinoma Risk in Egyptians
Source: Front Oncol. 2022 Jan 5;11:754104. doi: 10.3389/fonc.2021.754104 (PMC8766815; doi:10.3389/fonc.2021.754104)
Supplement: Supplementary file 1 [file DataSheet_1.doc]

**
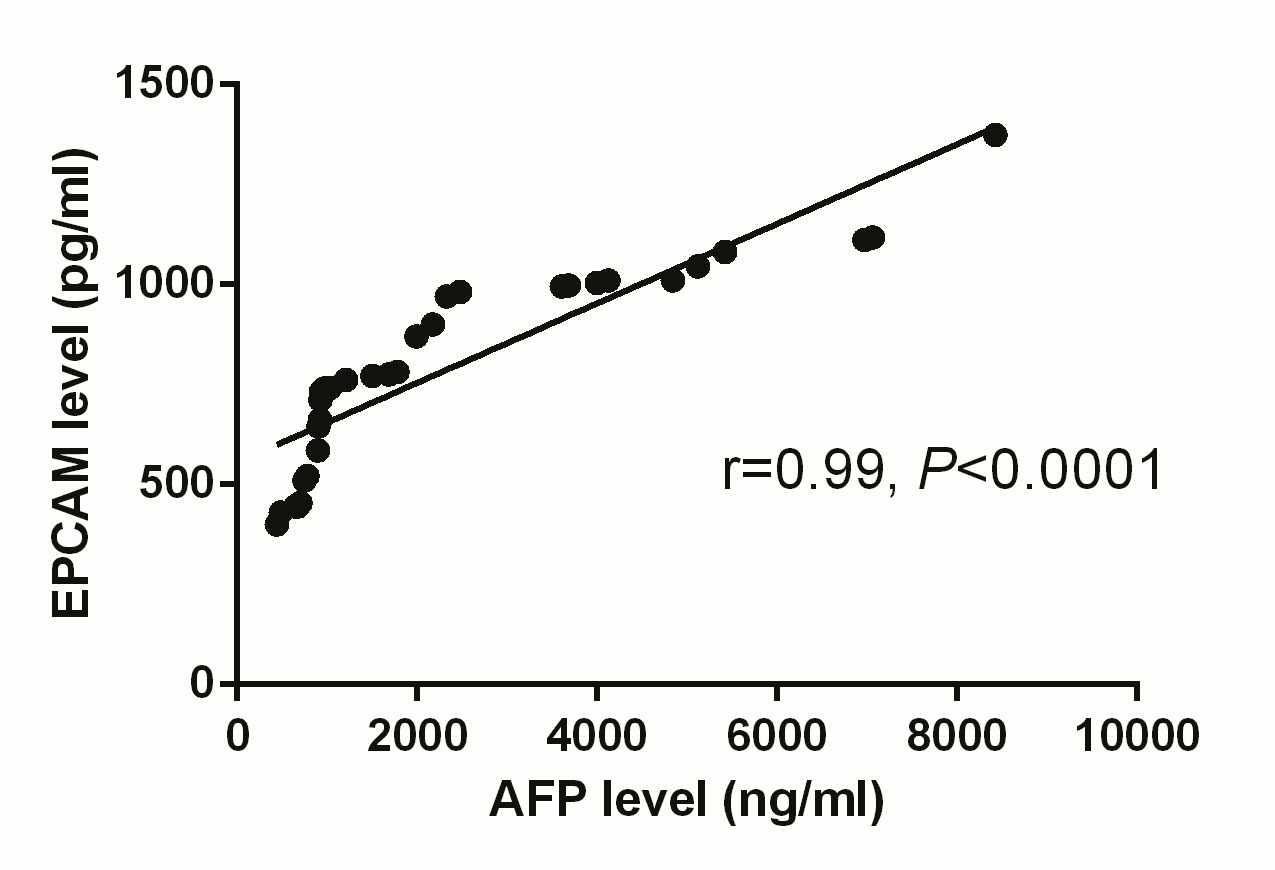
**

**Fig.S1. Spearman correlation analysis of AFP and EpCAM protein levels.**

**r: Spearman correlation coefficient**

| **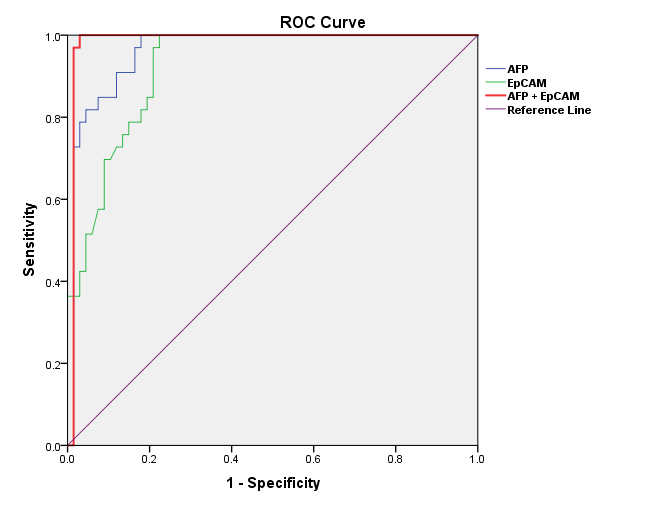** |
| --- |
| **AFP**, AUC= 0.961, CI= 0.92-0.99, *P*< 0.0001  **EpCAM**, AUC= 0.92, CI= 0.87-0.97, *P*< 0.0001  **AFP + EpCAM**, AUC= 0.99, CI= 0.95-1, *P*< 0.0001 |

**Fig.S2. ROC Curves of AFP, EpCAM, and Combined AFP and EpCAM.**

**AUC, area under the curve; CI, confidence interval**
