# Supplementary material for: rs62139665 Polymorphism in the Promoter Region of EpCAM Is Associated With Hepatitis C Virus-Related Hepatocellular Carcinoma Risk in Egyptians
Source: Front Oncol. 2022 Jan 5;11:754104. doi: 10.3389/fonc.2021.754104 (PMC8766815; doi:10.3389/fonc.2021.754104)
Supplement: Supplementary file 2 [file Table_1.doc]

| **Table S1. Clinical characteristics of hepatocellular carcinoma patients and hepatitis C virus infected patients** | | | |
| --- | --- | --- | --- |
| **Clinical characteristic** |  | **n (%)** |  |
| **HCC** | | |  |
| **Family history** | **Yes** | **3 (2.2%)** |  |
|  | **No** | **135 (97.8%)** |  |
| **Smoker** | **Yes** | **48 (34.8%)** |  |
|  | **No** | **90 (65.2%)** |  |
| **Anti-bilharzia treatment** | **Yes** | **85 (61.6%)** |  |
|  | **No** | **53 (38.4%)** |  |
| **Operation** | **Yes** | **62 (44.9%)** |  |
|  | **No** | **76 (55.1%)** |  |
| **Blood transfusion** | **Yes** | **20 (14.5%)** |  |
|  | **No** | **118 (85.5%)** |  |
| **Cachexia** | **Yes** | **3 (2.2%)** |  |
|  | **No** | **135 (97.8%)** |  |
| **Pallor** | **Yes** | **5 (3.6%)** |  |
|  | **No** | **133 (96.4%)** |  |
| **Jaundice** | **Yes** | **16 (11.6%)** |  |
|  | **No** | **122 (88.4%)** |  |
| **Lower limb edema** | **Yes** | **2 (1.4%)** |  |
|  | **No** | **136 (98.6%)** |  |
| **Liver size** | **Shrunken** | **8 (5.8%)** |  |
|  | **No** | **3 (2.2%)** |  |
|  | **Enlarged** | **41 (29.7%)** |  |
|  | **Average** | **86 (62.3%)** |  |
| **Spleen** | **No** | **51 (36.9%)** |  |
|  | **Enlarged** | **87 (63.1%)** |  |
| **Ascites** | **Yes** | **27 (19.6%)** |  |
|  | **No** | **111 (80.4%)** |  |
| **Number of focal lesion** | **1** | **76 (55.1%)** |  |
|  | **2** | **10 (7.2%)** |  |
|  | **Multiple** | **52 (37.7%)** |  |
| **Focal lesion site** | **Right lobe** | **104 (75.4%)** |  |
|  | **Left lobe** | **15 (10.8%)** |  |
|  | **Both lobes** | **19 (13.8%)** |  |
| **Alive or dead** | **Alive** | **46 (33.3%)** |  |
|  | **Dead** | **92 (66.7%)** |  |
| **HCV** | | |  |
| **Schistosoma serology test** | **Negative** | **128 (100%)** |  |
| **Liver Biopsy (HAI)** | **f1** | **38 (29.7%)** |  |
|  | **f2** | **77 (60.2)** |  |
|  | **f3** | **13 (10.1%)** |  |

Data are expressed as n (%).

HAI, Knodell histology activity index

f0, no fibrosis; f1, mild fibrosis; f2, moderate fibrosis; f3, severe fibrosis; f4, cirrhosis.

**Table S2. Liver ultrasound findings in the study groups**

|  | | **Control** | **HCV** | **HCC** |
| --- | --- | --- | --- | --- |
| **Liver Ultrasound** | **Normal** | **117 (100%)** | **60 (46.9%)** | **0 (0%)** |
|  | **Mild Hepatosplenomegaly** | **0 (0%)** | **8 (6.2%)** | **0 (0%)** |
|  | **Mild hepatomegaly** | **0 (0%)** | **2 (1.6%)** | **0 (0%)** |
|  | **Hepatosplenomegaly** | **0 (0%)** | **12 (9.4%)** | **0 (0%)** |
|  | **Homogenous hepatomegaly** | **0 (0%)** | **26 (20.3%)** | **0 (0%)** |
|  | **Fatty liver** | **0 (0%)** | **0 (0%)** | **0 (0%)** |
|  | **Coarse Liver** | **0 (0%)** | **13 (10.1%)** | **0 (0%)** |
|  | **Cirrhosis** | **0 (0%)** | **0 (0%)** | **138 (100%)** |
|  | **Bright hepatomegaly** | **0 (0%)** | **7 (5.5%)** | **0 (0%)** |
